# Cannabinoid type 2 receptor (CB2R) distribution in dermatomyositis skin and peripheral blood mononuclear cells (PBMCs) and in vivo effects of Lenabasum^TM^

**DOI:** 10.1186/s13075-021-02665-x

**Published:** 2022-01-04

**Authors:** Spandana Maddukuri, Jay Patel, De Anna Diaz, Kristen L. Chen, Maria Wysocka, Christina Bax, Yubin Li, Adarsh Ravishankar, Madison Grinnell, Majid Zeidi, Nithin Reddy, Josef Symon S. Concha, Muhammad M. Bashir, Joyce Okawa, Barbara White, Victoria P. Werth

**Affiliations:** 1 Department of Dermatology, Corporal Michael J. Crescenz Veterans Affairs Medical Center, Philadelphia, PA USA; 2 Department of Dermatology, Perelman School of Medicine, University of Pennsylvania, Philadelphia, PA USA; 3 Department of Pathology, SUNY Downstate Health Sciences University, Brooklyn, NY USA; 4 Department of Medicine, Division of Dermatology, Albert Einstein College of Medicine and Montefiore Medical Center, Bronx, NY USA; 5 Corbus Pharmaceuticals, Inc., Norwood, MA USA

**Keywords:** CB2R, Dermatomyositis, Image mass cytometry, Lenabasum, Dendritic cells

## Abstract

**Background:**

Lenabasum is a cannabinoid type 2 receptor (CB2R) reverse agonist that demonstrates anti-inflammatory effects in vivo and in vitro in dermatomyositis (DM) and is currently being investigated for therapeutic potential. The purpose of our study is to investigate CB2R distribution as well as the effects of lenabasum in DM.

**Methods:**

Immunohistochemistry staining (IHC) was utilized to examine immune cell and cytokine production changes in lesional DM skin biopsies from lenabasum and placebo-treated patients. CB2R expression in various immune cell populations within DM skin was investigated with image mass cytometry (IMC), whereas flow cytometry elucidated CB2R expression in DM peripheral blood mononuclear cells (PBMCs) as well as cytokine production by CB2R-expressing cell populations.

**Results:**

After 12 weeks of lenabasum treatment, IHC staining showed that CD4+ T cells, CB2R, IL-31, IFN-γ, and IFN-β cytokines were downregulated. IFN-γ and IFN-β mRNA decreased in lesional DM skin but not in PBMCs. IMC findings revealed that CB2R was upregulated in DM lesional skin compared to HC skin and DM PBMCs (*p*<0.05). In DM skin, CB2R was upregulated on dendritic cells, B cells, T cells, and macrophages while dendritic cells had the greatest expression in both DM skin and PBMCs (*p*<0.05). These CB2R+ cells in the skin produce IL-31, IL-4, IFN-γ, and IFN-β.

**Conclusion:**

Our findings of differential CB2R expression based on location and cell type suggest modes by which lenabasum may exert anti-inflammatory effects in DM and highlights dendritic cells as potential therapeutic targets.

**Supplementary Information:**

The online version contains supplementary material available at 10.1186/s13075-021-02665-x.

## Introduction

Dermatomyositis (DM) is a rare chronic systemic autoimmune disease that primarily affects the skin, muscle, and lungs [1]. Skin disease is observed in virtually all DM patients, whether they have amyopathic or classic DM [2]. Cutaneous features of DM include a heliotrope rash, Gottron papules, shawl sign, and V-neck sign, among other skin findings [3]. Pruritus is also known to be a common clinical feature [4, 5]. DM skin lesions substantially reduce patients’ quality of life (QoL) because they are associated with visible disfigurement, persistent pruritus, and photosensitivity [5]. Antimalarials are first-line therapy for DM. However, they are often ineffective and cause adverse drug reactions [6–8]. Systemic therapies for antimalarial-refractory DM include glucocorticoids, immunosuppressives, and intravenous immunoglobulin (IVIG) but are associated with numerous side effects [8]. The decreased QoL of DM patients combined with the inadequacy of current treatments highlight the therapeutic need for effective and safer treatment options.

Lenabasum, a nonpsychoactive, nonimmunosuppressive cannabinoid type 2 receptor (CB2R) reverse agonist, is currently being investigated as a potential treatment option for DM. Cannabinoid receptors are G-protein coupled receptors with two major subtypes: cannabinoid type 1 receptors (CB1R) expressed mainly in the central (CNS) and peripheral nervous system (PNS), and CB2R primarily distributed throughout immune and hematopoietic cells, myocytes, epithelial cells, fibroblasts, osteoblasts, skin keratinocytes, and the PNS [9]. Lenabasum is a preferential CB2R agonist with a 12.3-fold greater affinity for CB2R than CB1R [10] and inefficient penetration into the CNS [10–12], resulting in its nonpsychoactive effects. Lenabasum also activates peroxisome proliferator-activated receptor γ (PPARγ), a nuclear hormone receptor, leading to changes in the transcription of target genes involved in cell differentiation and inflammation, either downstream of CB2R activation or via direct binding [13, 14]. This may also contribute to the suppression of inflammatory cytokine production.

Exogenous synthetic cannabinoids represent a novel approach to modulating inflammation, as the activation of CB2R has been shown to reduce several key pro-inflammatory cytokines implicated in DM, including type I IFNs, IL-1, IL-6, IL-17, and TNF-α [15–19]. Furthermore, clinical trials of orally administered lenabasum found no marijuana-like psychoactive effects or serious adverse effects [20]. Previously in our lab, we demonstrated lenabasum’s anti-inflammatory effects on DM through in vitro studies. We investigated lenabasum’s effect on TNF-α, IFN-α, and IFN-β production by peripheral blood mononuclear cells (PBMCs) from DM blood and found that moderate and high concentrations of lenabasum suppressed TNF-α secretion, while all lenabasum doses (low, moderate, and high) suppressed IFN-α and IFN-β production [21]. We also found that lenabasum significantly suppressed IL-31, a cytokine implicated in the pruritus of DM, and IL-4 production by PBMCs [4].

However, we had not previously explored the distribution of the CB2R among various immune cells within the scope of DM or examined differences in CB2R expression between healthy controls (HC) and DM patients. Furthermore, differences between CB2R expression in DM skin and blood remain largely unknown. Although some of lenabasum’s in vitro effects on cytokine production have been demonstrated through previous studies, further knowledge of CB2R’s distribution in DM is crucial to understanding lenabasum’s mechanism of action. Additionally, this understanding of CB2R distribution in DM may provide further insight into DM pathogenesis by identifying cells that are modulated by lenabasum, given significant clinical improvement with treatment. This insight may also be applicable to other diseases driven by cell types affected by lenabasum. The purpose of our study is to use flow cytometry and the novel technique of image mass cytometry (IMC) to elucidate CB2R distribution in DM skin and blood to better understand the targets of lenabasum.

## Results

### CB2R expression is increased in dermatomyositis skin compared to healthy control skin

**Fig. 1:**
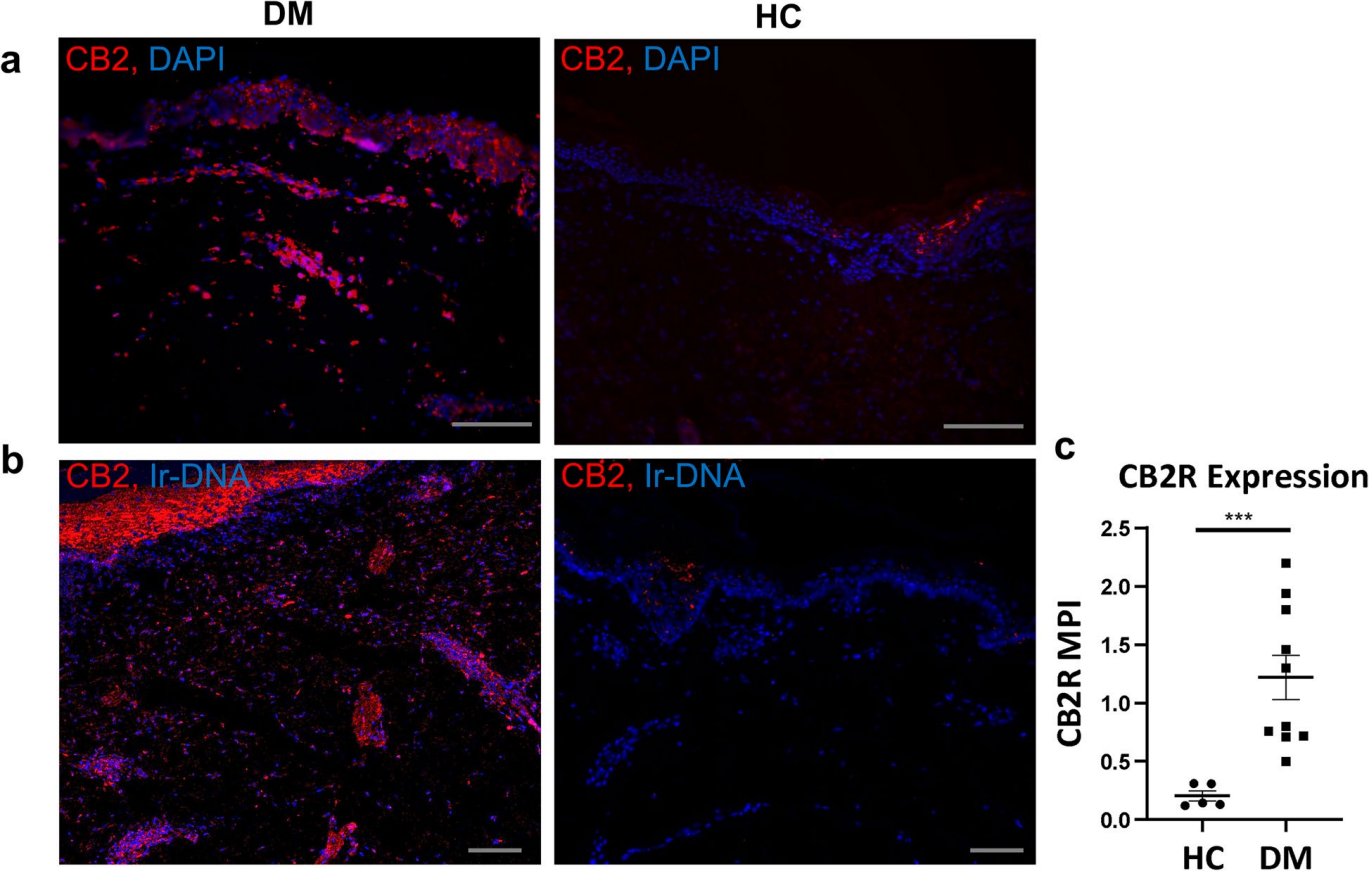
CB2R upregulation in dermatomyositis compared to healthy control. Skin biopsies stained via **a** immunofluorescence demonstrate CB2R (red) expression in DM skin compared to HC skin. Nuclei stained with dapi. **b** IMC demonstrates CB2R (red) expression in DM skin compared to HC skin. Nuclei stained with Ir-DNA. **c** Quantification of IMC CB2R expression in DM lesional skin compared to HC skin, excluding epidermis. Scale bars (gray) = 100μm. Graph shows median **±** IQR. ***p<0.001. DM, dermatomyositis; HC, healthy control; IMC, image mass cytometry, iridium intercalator (Ir-DNA); MPI, mean pixel intensity

To evaluate CB2R expression in DM skin, we first utilized immunofluorescence (IF) staining and observed increased expression of the CB2R marker in DM skin compared to HC skin (Fig. 1a, red). We then utilized IMC to further characterize the inflammatory infiltrates of 5 HC and 10 DM patients with moderate-severe disease. Through IMC, we confirmed increased expression of the CB2R marker in DM skin compared to HC Skin (Fig. 1b, red). Statistical analysis comparing mean pixel intensity (MPI) demonstrated IMC CB2R expression in DM lesional skin to be significantly greater than in HC skin (*p*<0.001, Fig. 1c).

### Dendritic cells and B cells demonstrate increased expression of CB2R among immune cell populations in DM inflammatory skin infiltrate

**Fig. 2:**
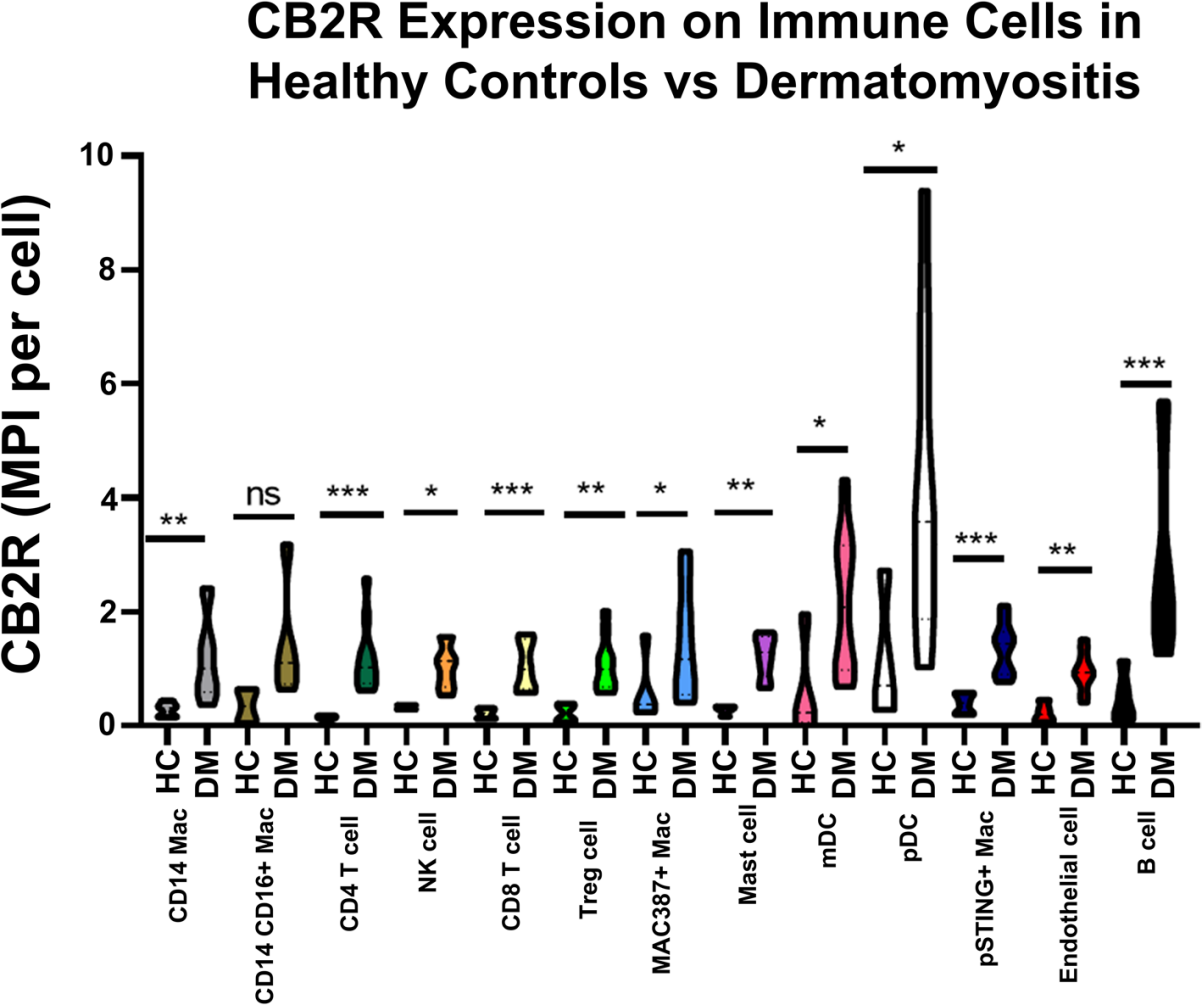
CB2R distribution among immune cells in DM and HC skin. DM and HC skin cells were stained via IMC. IMC shows the distribution of CB2R mean pixel intensity (MPI) per cell in DM and HC skin for the following immune cell populations: CD14, CD14+CD16+, CD4+, CD56+, CD8+, mDC, pDC, FOXP3+, MAC387+, mast cell, pSTING+, endothelial, and CD20+. Graph shows median **±** IQR. *p<0.05, **p<0.01, ***p<0.001. IMC, image mass cytometry; MPI, mean pixel intensity; mDC, myeloid dendritic cell; pDC, plasmacytoid dendritic cell

We further utilized IMC to evaluate CB2R expression among 13 unique immune cell populations in DM inflammatory infiltrate and quantified expression based on MPI. The immune populations are listed here in decreasing order according to percent composition of the inflammatory infiltrate: CD14+ macrophages (Mac), CD11c+ myeloid dendritic cells (mDCs), CD14+CD16+ Mac, CD4+ T cells, MAC387+ Mac, mast cells, CD8+ T cells, FOXP3+ T cells, pSTING+ Mac, BDCA2+ pDCs, CD56hi NK cells, and B cells [22]. We found dendritic cells and B cells express the greatest percentage of CB2R among immune cell populations in the DM inflammatory infiltrate. CB2R expression in pDCs was significantly greater when compared to all other immune cells (*p*<0.05, Fig. 2) except mDCs and B cells (*p*>0.05). B cells expressed significantly increased CB2R compared to CD14+ macrophages, CD14+CD16+ macrophages, T cells, and NK cells (*p*<0.05) with no difference compared to CB2R expression on pDCs, mDCs, and pSTING+ macrophages (*p*>0.05).

Comparison of CB2R expression in DM versus HC immune cell populations revealed a statistically significant increase in CB2R expression on all cell populations (*p*<0.05, Fig. 1b) except CD14+CD16+ Mac (Fig. 2, *p*=0.053) in DM lesional skin. CB2R expression on DM lymphocytes and dendritic cells in the skin was further explored using flow cytometry on eluted cells from eight DM lesional skin biopsies; two biopsies were stained with the dendritic cells panel, two were stained with the lymphocyte panel, and four biopsies were stained with both panels. Flow cytometry results corroborated IMC findings and showed a trend to a greater percentage of pDCs (60.3% ± 70.98%) expressing CB2R compared to mDCs (28.10% ± 35.6%) and CD4 T cells (26.0% ± 24.0%), although not statistically significant. Again, no difference was noted between pDC and mDC expression of CB2R.

### CB2R expression is greater in DM skin compared to DM PBMCs

**Fig. 3:**
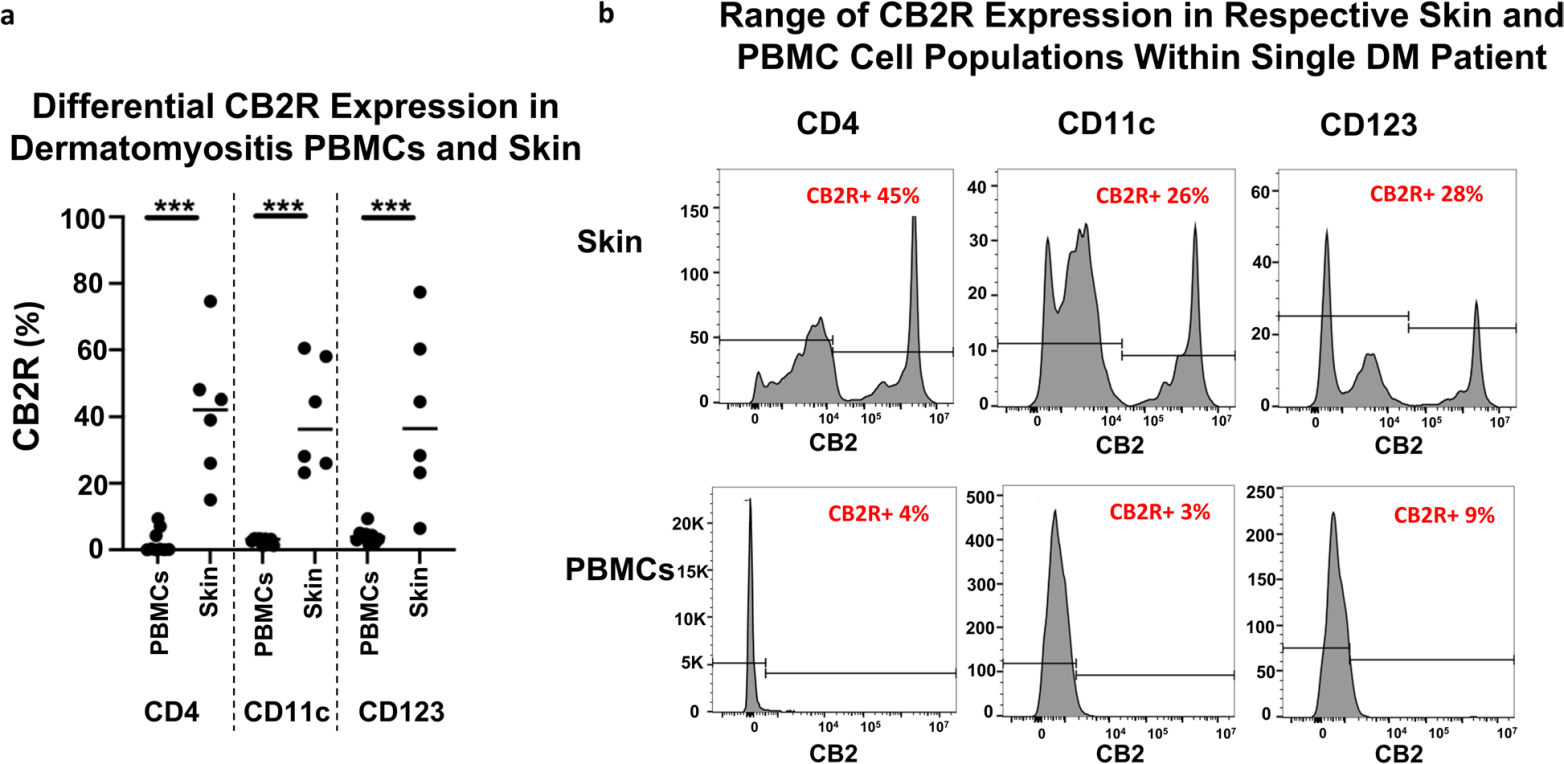
CB2R distribution among DM skin and DM PBMCs via flow cytometry. DM and HC peripheral blood PBMCs and skin cells isolated and stained via flow cytometry. **a** Percentage of CB2R expression in CD4+ T cells, CD11c+ mDCs, and CD123+ pDCs compared between DM peripheral blood and skin. **b** CB2R expression in skin and peripheral blood samples of a single patient shown with respect to CD4+ T cells, CD11c+ mDCs, and CD123+ pDCs. Graph shows the median. ***p<0.001. DM, dermatomyositis; HC, healthy control; mDC, myeloid dendritic cells; PBMC, peripheral blood mononuclear cell; pDC, plasmacytoid dendritic cell

Given the significant expression of CB2R in DM skin, we compared expression in DM peripheral blood versus DM lesional skin via flow cytometry. We isolated the PBMCs and lesional skin cells of 6 HC and 6 DM patients and found higher overall expression of CB2R in eluted DM skin cells (28.10% ± 40.05%) compared to DM PBMCs (2.097% ±3.18%) (*p*<0.0001). When stratified by cell type, DM CD4+ T cells, mDCs (CD11c), and pDCs (CD123) from the skin had significantly greater CB2R expression than their peripheral blood counterparts as well (*p*<0.05, Fig. 3a, b). Fluorescent minus one (FMO) controls and gating strategy for CB2R positivity are depicted in Fig. S2.

### CB2R expression is greater on PBMC dendritic cells compared to lymphocytes

**Fig. 4:**
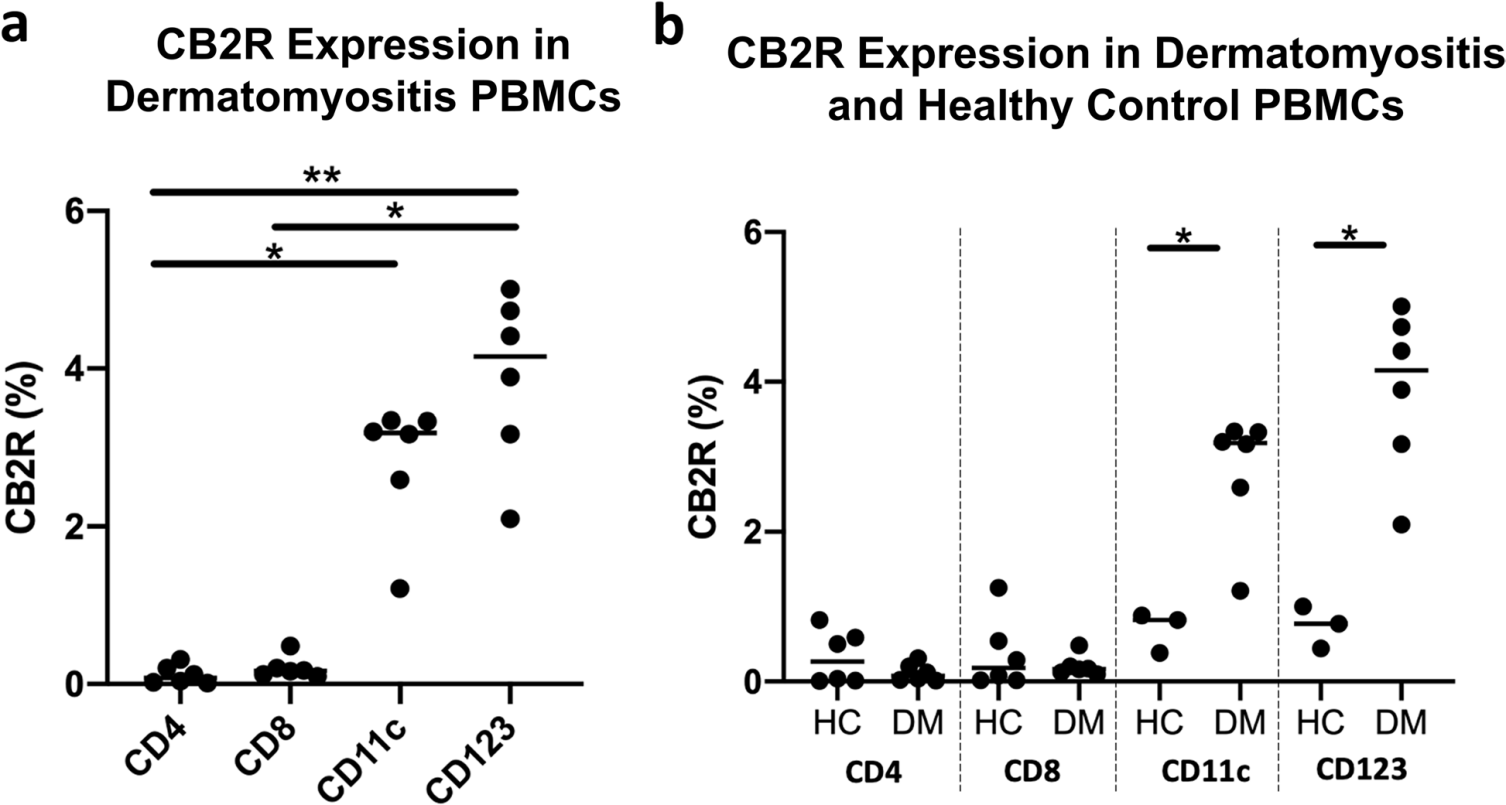
CB2R distribution among DM and HC PBMC populations via flow cytometry. DM and HC PBMCs and skin cells were isolated and stained via flow cytometry. **a** Percentage of CB2R+ cells among DM CD4+ T cells, CD8+ T cells, CD11c+ mDCs, and CD123+ pDCs. **b** Percentage of CB2R expression among CD4+ T cells, CD8+ T cells, CD11c+ mDCs, and CD123+ pDCs is compared between HC and DM PBMCs. Graph shows median. *p<0.05, **p<0.01. DM, dermatomyositis; HC, healthy control; mDC, myeloid dendritic cells; PBMC, peripheral blood mononuclear cell; pDC, plasmacytoid dendritic cell

We evaluated CB2R expression on dendritic cells in the peripheral blood and compared it to that of lymphocyte populations. CB2R expression was significantly greater on mDCs and pDCs when compared to CD4+ T cells (*p*<0.01 and *p*<0.05, respectively, Fig. 4a) and on pDCs compared to CD8+ T cells (*p*<0.05, Fig. 4a). When CB2R expression on CD4+ T cells, CD8+ T cells, mDCs, and pDCs was compared between HC and DM patients, significantly greater expression was noted on DM mDCs (*p*<0.05) and pDCs (*p*<0.05) compared to HC mDCs and pDCs (Fig. 4b). No significant differences in CB2R expression were noted between DM and HC CD4+ and CD8 T+ cells (Fig. 4b).

### CD4+ T, CD11c+, and CD123+ cells expressing CB2R in DM skin produce IL-31, IL-4, IFN-γ, and IFN-β

**Fig. 5:**
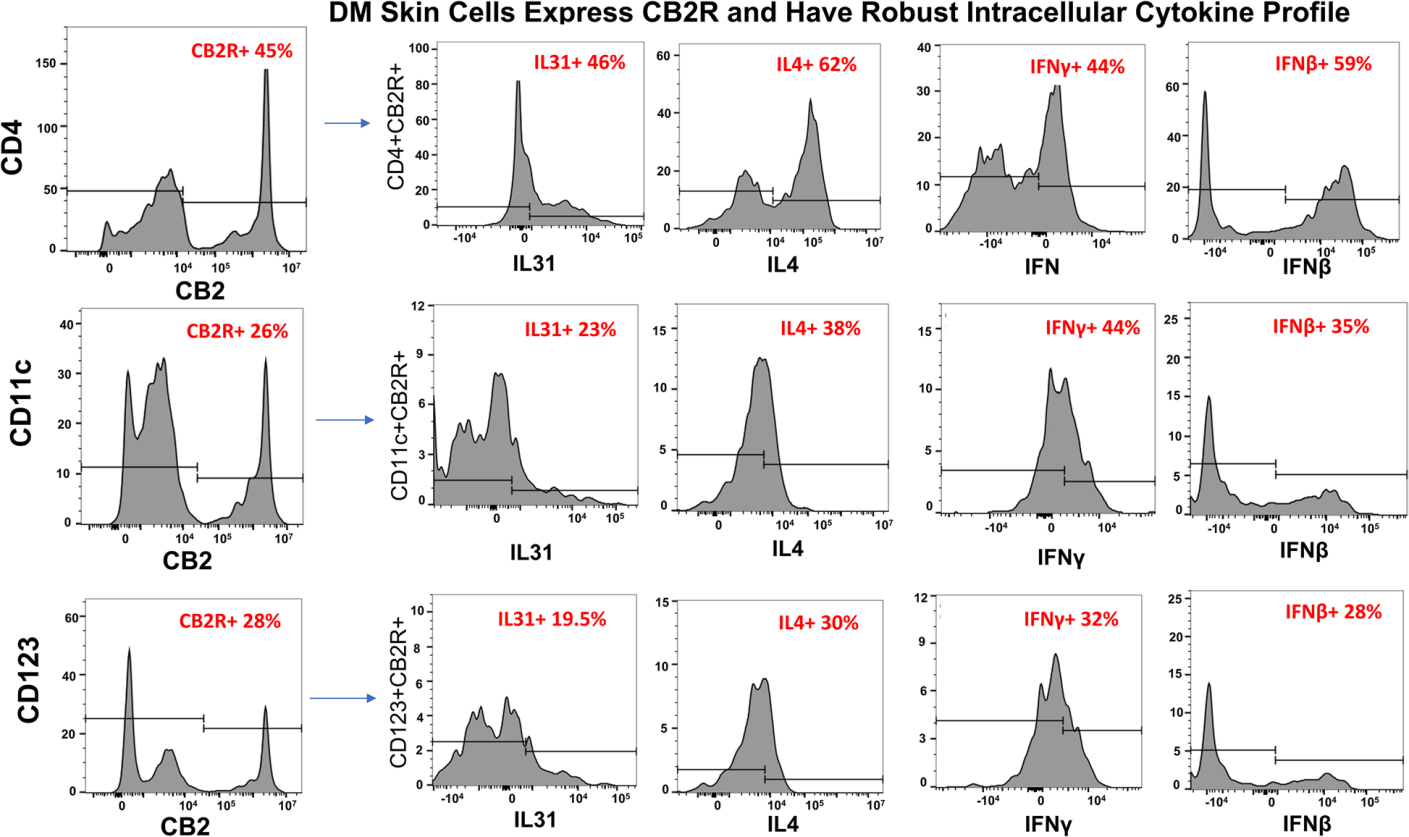
Robust intracellular cytokine profile of DM skin cells expressing CB2R. DM skin cells were isolated and stained via flow cytometry. Flow cytometry identifies a population of CD4+ T cells, CD11c+ mDCs, and CD123+ pDCs that express CB2R in DM skin. Flow cytometry quantifies IL-31, IL-4, IFN-γ, and IFN-β produced by each population. DM, dermatomyositis; mDC, myeloid dendritic cells; pDC, plasmacytoid dendritic cell

Flow cytometric analysis of the lymphocyte and dendritic cell populations eluted from DM lesional skin demonstrated that the CD4 T+ cells, mDCs, and pDCs robustly express CB2R (Fig. 5). When CB2R expression among these populations was further stratified by intracellular cytokine production (Fig. 5), all three populations were found to produce IL-31, IL-4, IFN-γ, and IFN-β. CD4+ lymphocytes expressing CB2R were shown to produce the largest percentage of IL-31. Both dendritic cells and CD4 T+ cells expressing CB2R produced IL-4, IFN-γ, and IFN-β. These results were confirmed in three patient skin biopsies, with FMO controls and gating strategy are depicted in Fig. S3.

### Lenabasum reduces CD4+ T cell population and downregulates Type 1 IFN expression in lesional DM skin

**Fig. 6:**
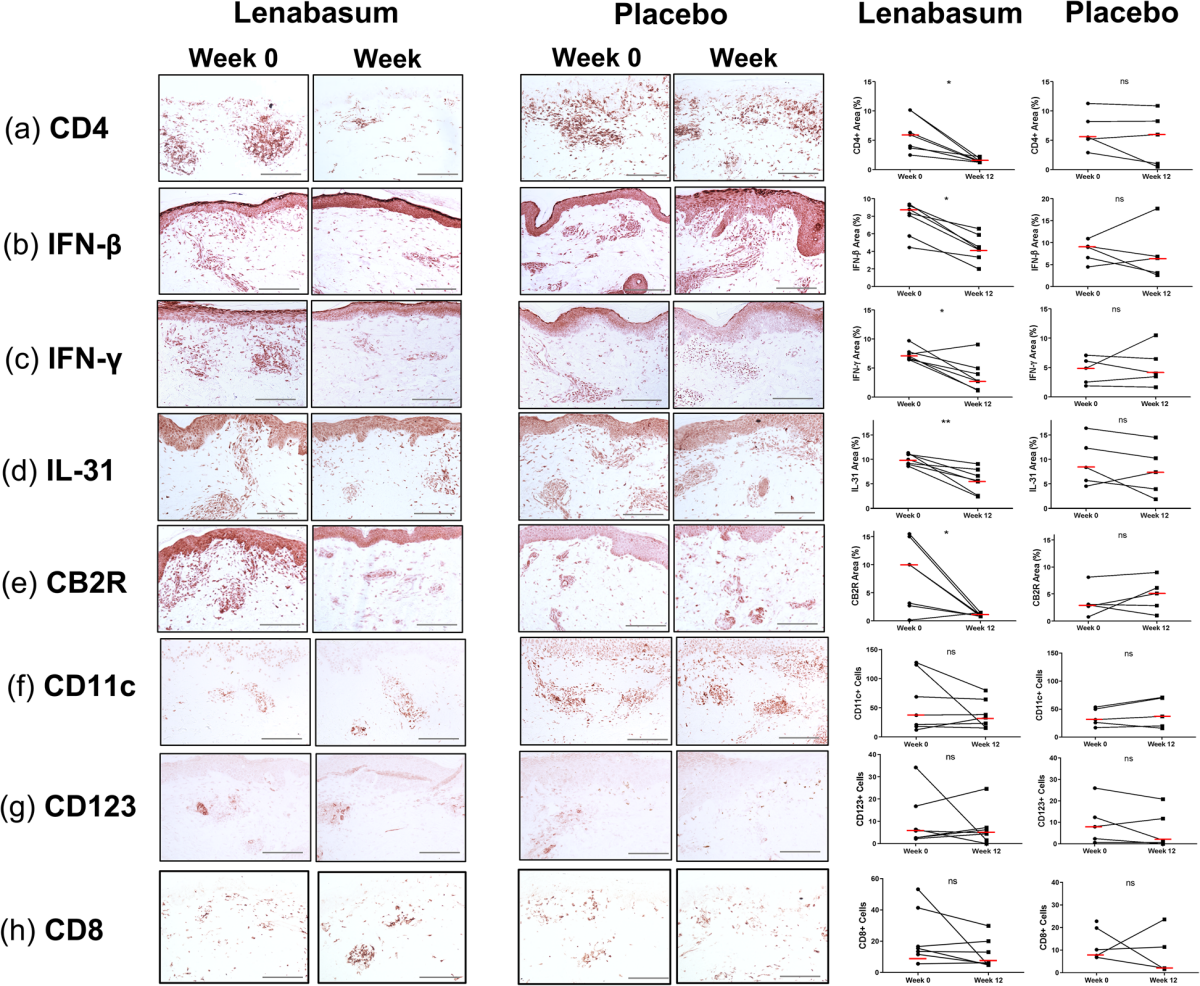
Cytokine and cell expression in DM lesional skin treated with Lenabasum versus placebo. Skin biopsies from lesional DM skin stained via immunohistochemistry for **a** CD4+ cells, **b** IFN-β, **c** IFN-γ, **d** IL-31, **e** CB2R, **f** CD11c+ cells, **g** CD123+ cells, and **h** CD8+ cells at week 0 and week 12 following treatment with either lenabasum or placebo. Representative IHC images are pictured at ×20 objective magnification. Quantification of cell counts and percentage of area stained positive are pictured adjacently. Cells were counted in five non-overlapping ×40 fields and averaged. Percentage of area stained positive was quantified in one ×20 field. Epidermis excluded. Graph shows the median. *p<0.05

Skin biopsies and PBMCs from DM patients in the phase 2 randomized, double-blind, placebo-controlled trial were investigated at baseline and after 12 weeks of treatment with oral lenabasum. Immunohistochemistry (IHC) staining of skin biopsies revealed decreased CD4+, IFN-β, IFN-γ, IL-31, and CB2R protein expression in lenabasum-treated subjects at 12 weeks (*p*<0.05, Fig. 6), with no differences in the placebo group (*p*>0.05, Fig. 6a–e). There were no significant changes in the number of mDCs, pDCs, or CD8 T cells (*p*>0.05, Fig. 6f–h). RNA extraction and quantitative real-time PCR of skin additionally found IFN-β and IFN-γ mRNA decreased significantly in the lenabasum-treated group (*p*<0.05, Fig. S1) but not in the placebo group, with no differences found for IL-31 and IL-4 mRNA (*p*>0.05, Fig. S1). PBMC mRNA was similarly compared in the lenabasum-treated group and placebo group; however, no significant differences were found for IFN-β, IFN-γ, IL-31, and IL-4 mRNA (*p*>0.05, Fig. S1).

## Discussion

The therapeutic potential of the CB2R is increasingly being recognized for its immunomodulatory properties. Several studies have confirmed the anti-inflammatory effects of CB2R activation in inflammatory diseases through murine models [23–29]. Certain CB2R agonists have been shown to limit chemotaxis and migration of immune cells such as monocytes and dendritic cells [30–32]. The anti-inflammatory properties of CB2R agonists, including lenabasum, are now being investigated in clinical trials [33, 34].

Here we show lenabasum modulated the immune environment within 12 weeks via a decrease in CD4+ T cells and pathogenic cytokines in DM such as IFN-β, IFN-γ, and IL-31 within the skin. Importantly, such differences were not observed in the mRNA of PBMCs (Fig. S1), suggesting a CB2R-based specificity. The lack of decrease in IL-4 and IL-31 mRNA in Lenabasum -treated skin may be attributed to CB2R-mediated post-transcriptional modifications such as mRNA stability, protein translation efficiency, protein half-life modification, and induction of inhibitors, as IL-4 and IL-31 protein levels did decrease. There are no known studies that describe CB2R distribution within the scope of DM and so we further investigated CB2R distribution using QRT-PCR, IHC, IF, IMC, and flow cytometry in HC and DM patients to identify the target immune cell populations and possible mechanisms of action of lenabasum.

The CB2R is largely activation-dependent and is expressed on almost all immune cells; it regulates migration, proliferation, and effector functions [35]. Previous studies on rat models have demonstrated that a setting of inflammation leads to CB2R upregulation [36, 37]. To date, in humans, much of the data regarding CB2R expression has been obtained via PBMCs of healthy donors [38, 39]. However, based on the differential cytokine changes in skin compared to PBMCs after lenabasum treatment and due to the receptor’s activation-dependent nature, we suspected the distribution of CB2R expression in DM may differ in circulation versus skin depending on local disease activity and inflammation.

Consistent with our hypothesis that CB2R may be upregulated in the setting of local inflammation in DM, we found CB2R expression was significantly increased in lesional DM skin compared to normal HC skin. This effect is also evident by the increased CB2R expression in DM skin compared to blood, possibly due to increased production of local inflammatory cytokines [40]. Additionally, our study showed CB2R downregulation with lenabasum treatment, as CB2R expression decreased significantly in lesional DM skin following 12 weeks of treatment with lenabasum and no change occurred in the placebo group. Through IMC we observed increased CB2R expression on skin immune cells, particularly, dendritic cells and B cells. Lenabasum treatment decreased this CB2R expression in lesional DM skin following 12 weeks of treatment, which was not observed with placebo. This reduction in CB2R expression may be due to the effects of the reverse agonist resulting in downregulation of pathologic CB2R expression at a cellular level, as occurs with tachyphylaxis, or alternatively on a global level due to decreased infiltrating cells. Long-term suppression of CB2R expression may be desirable as this may reduce inflammatory relapse, but the specific mechanisms require further investigation.

CB2R expression on dendritic cells has been demonstrated previously using blood-derived dendritic cells [41], but here we show upregulated *in vivo* expression in the skin through IMC. Our observations of hierarchical CB2R expression on pDCs, B cells, mDCs, then macrophages, and finally, T cells is similar to patterns observed in secondary lymphoid tissue [39] with the major finding in our study suggesting dendritic cells express the highest density of CB2R per cell.

Although pDCs were identified as the major expressor of CB2R, it is important to note that our study found no difference in CB2R expression between mDCs and pDCs, suggesting the equal importance of both dendritic cell populations when considering lenabasum’s effect. Furthermore, pDCs are significantly less prevalent than CD4+ T cells and mDCs in DM skin [42]. It is thus possible that CD4+ T cells and mDCs express more CB2R overall, despite fewer CB2R expressed per cell, than the relatively rare pDCs and B cells populations in DM skin. This expression pattern highlights the possible use of CB2R agonists in modulating T cell and dendritic cell functions, especially those involving the type I IFN response, which has been implicated in DM pathogenesis [43]. One study has demonstrated the suppressive capabilities of CB2R selective agonists on pDC production of IFN-α and TNF-α [21, 44], which are elevated in autoimmune conditions such as systemic lupus erythematosus and may also be implicated in DM pathogenesis [43, 45]. Increased CB2R expression on mDCs and pDCs in DM skin may be a particularly important finding for other cutaneous autoimmune diseases in which dendritic cells contribute to the type I IFN axis. These findings suggest a potentially powerful therapeutic target, as dendritic cells are known to amplify the adaptive immune response via activation and proliferation.

Despite the extremely low prevalence of B cells in DM skin, our study found that they express CB2R. Since autoantibody production is a critical component of DM pathogenesis and previous studies demonstrated CB2R’s regulation of B cell migration, memory response, and antibody production [35, 46], CB2R expression in this population warrants further investigation. The increased CB2R expression in this population may serve as another modifiable therapeutic axis for targeting autoimmune memory responses in DM, despite being relatively rare in DM skin.

The cytokines decreased with lenabasum treatment (IFN-β, IFN-γ, and IL-31) were all found in CB2R expressing CD4+ T cells, mDCs, and pDCs. This suggests lenabasum may modulate disease activity via its effects on the CB2R expressed on multiple immune cell populations, which include downregulation of multiple cytokines in a single cell, particularly IFN-β, which is thought to drive disease activity [43]. Lenabasum treatment also decreased the number of infiltrating skin CD4+ T cells, which may be due to CB2R-mediated downregulation of cell migration; this could serve as another mechanism by which cytokine production may be suppressed. Lenabasum does not appear to alter mDC or pDC cell infiltration, but may still influence their cytokine production. The effects of lenabasum on cytokine production and migration of these populations will be investigated in the future.

This study complements previous reports of mDCs and CD4 T cells producing IFN-β and constituting a high percentage of the DM infiltrate by demonstrating upregulation of CB2R [22]. This upregulation of CB2R may lead to increased cell migration and cytokine production, resulting in skin lesions. The increased expression on dendritic cells suggests an amplification effect whereby an initial insult (viral, ultraviolet light exposure, or trauma) may upregulate dendritic cell CB2R expression, which, in turn, promotes recruitment and stimulation of secondary immune cells. Individuals may also be predisposed to CB2R upregulation via the effects of other medications, herbal supplements, or genetic predispositions. This local upregulation of CB2R expression provides evidence for trials of topical or injectable lenabasum as it may suppress locally inflammatory dendritic cells while permitting central regulatory cell infiltration. Knowing the distribution of CB2R is essential for identifying patients and diseases targetable by lenabasum, as skin biopsies may demonstrate CB2R dominance, suggesting a predictable response. Lenabasum's effects in other inflammatory diseases mediated by dendritic cells and B cells should also be investigated, as it may offer therapeutic benefit in these conditions.

Our study is not without limitations. The small sample size of IMC studies warrants further investigation, as CB2R expression may differ in rare human skin populations like B cells. As human dendritic cells have been identified as the center of a dysregulated autoimmune response in DM and cutaneous lupus erythematosus [22, 42, 47], future studies should focus on the effects of CB2R modulation in these cells. Moreover, the effects of lenabasum on PPARγ need to be further investigated, as this pathway has significant effects on immune cell function and was not evaluated in this study.

## Conclusion

In this study, we have identified effects of lenabasum in skin lesions and discovered novel patterns of expression of CB2R in DM skin and blood by which the drug may act. Lenabasum was found to downregulate CD4 T cells, CB2R, and cytokines contributing to DM pathogenesis in the skin. Upregulation of CB2R on immune cells in skin and blood, and in particular dendritic cells highlight targets of lenabasum. By demonstrating CB2R expression on key immune cells and highlighting effects on pathways we may better direct this emerging therapy to identify DM patients with susceptible lesions. Knowledge of CB2R distribution and the effects of Lenabasum may also translate to other autoimmune skin diseases and will be of importance to the field of dermatology as this drug is further developed.

## Materials and methods

### Patients

All patients and healthy controls (HC) were recruited from the Department of Dermatology at the University of Pennsylvania (Institutional Review Board approved). All subjects signed informed consent before participating in this study. All DM patients were diagnosed by VPW using either European League Against Rheumatism/American College of Rheumatology (ACR) or Sontheimer criteria. In our IHC and mRNA study, lesional skin from DM patients with moderate-severe, refractory, skin-predominant DM on stable standard-of-care treatments was recruited for a double-blind, placebo-controlled, randomized trial. Demographics are detailed in Table S1. These patients initially received lenabasum 20 mg daily for 4 weeks, then subsequently received 20 mg twice daily for an additional 8 weeks. Biopsies were obtained at weeks 0 and 12. In the remaining studies, lesional skin biopsies and peripheral blood samples were obtained from DM patients in a longitudinal database who were not enrolled in the trial and were not necessarily refractory to standard of care therapy. In our IMC analysis, lesional skin biopsies were obtained from 5 HC and 10 DM patients with newly diagnosed moderate-severe DM. Demographics are detailed in Table S2. In our flow cytometry analysis, 7 lesional skin biopsies were obtained from DM patients. Demographics are detailed in Table S3. Blood samples were obtained from a total of 11 HC and 17 DM patients and certain HC and DM samples were stained with multiple FC panels. Demographics are detailed in Table S4.

### Immunohistochemical staining

See Supplementary methods.

### Immunofluorescence staining

See Supplementary methods.

### Image mass cytometry

#### Antibody conjugation and image acquisition

Prior to the processing of tissue, antibodies were conjugated to different metal isotopes using the Maxpar Antibody Labeling Kit (Fluidigm, San Francisco, CA). Tissue sections were incubated in a cocktail of 17 metal-conjugated antibodies (listed in Table S6) and processed with the Hyperion Imaging System (Fluidigm, San Francisco, CA). Regions of interest (ROI) up to 2 × 1 mm were ablated at a frequency of 200 Hz. For further details, see Supplementary methods.

#### Image processing and data analysis

The resulting MCD image files were exported to 32-bit TIFF files using MCD Viewer^TM^ (Fluidigm). Cell segmentation was performed using a nuclear app-based algorithm in Visiopharm (Westminster, CO). The resulting mask and TIFF files were imported into histoCAT (Fluidigm), where per object MPI data was generated. The phonograph algorithm was used for unsupervised clustering of cell populations. MPI for different cell populations and related heatmaps were gathered using histoCAT. Colocalization images were created using ImageJ.

### RNA extraction and quantitative real-time reverse transcriptase polymerase chain reaction (QRT-PCR)

See Supplementary methods.

### Flow cytometry

#### PBMC isolation

PBMCs were isolated by density gradient over Ficoll-Hypaque (Sigma-Aldrich, St. Louis, MO) by standard procedures isolated PBMCs at 1 × 10^6^ cells/mL were cultured in RPMI culture medium supplemented with 10% fetal bovine serum, 1% L-glutamine, and 1% penicillin-streptomycin.

#### Elution of skin cells

Biopsies were minced and digested overnight in 0.08% Trypsin and subsequently digested in 0.125 mg/mL Liberase TL (Sigma-Aldrich) in DMEM media for 2 h at 37°C the following day. Samples were then mashed through 70-mm cell strainers and washed with a medium. Eluted PBMCs were then counted and samples with 1 × 10^6^ cells/mL were selected for culture and staining.

#### Stimulation of cells, staining, and acquisition of data

Cells to be stained with lymphocyte antibodies were stimulated with a cell activation cocktail (2 mg/mL) containing phorbol myristate acetate/ionomycin/Brefeldin A (Biolegend-423303, San Diego, CA) for 4 h at 37°C. Cells to be stained with dendritic cell antibodies were stimulated with R848 (1ug/mL) (Invivogen- tlrl-r848, San Diego, CA) and Brefeldin A (1mg/mL) (Biolegend-42060) for 5 h at 37°C. Detailed staining protocol is included in Supplementary methods. Subsequent to staining, single-cell suspensions underwent flow cytometric analysis on a BD FACS Canto (5 samples) or BD FACS Symphony (2 samples) (BD Biosciences, San Jose, CA) and were analyzed with the FlowJo software (BD Biosciences). The intensity of the staining was measured by percent of parent population, and gating was determined by the mean fluorescence intensity (MFI) values. To distinguish between specifically stained cells and background fluorescence, we used appropriate controls, including unstimulated samples and fluorescence-minus-one controls [48]. We defined this MFI value as a cut-off and considered a cell as positive for a given marker if their MFI exceeded this cut-off value and cells with MFI values below this cut-off value were referred to as cytokine-negative cells. Further data regarding gating process can be found in the Supplementary methods.

### Statistical analysis

The Wilcoxon signed-rank test, Mann-Whitney test, Kruskal-Wallis test, and Bonferroni’s multiple comparisons test were used. Statistical analysis was performed using GraphPad Prism V8 software (GraphPad Software, Inc., La Jolla, CA).

## Supplementary Information

**Additional file 1.** Supplementary methods

## Data Availability

Datasets related to this article are available upon request to the corresponding author.

## Abbreviations

DM: Dermatomyositis
CB2R: Cannabinoid type 2 receptor
CB1R: Cannabinoid type 1 receptor
mDC: Myeloid dendritic cell
pDC: Plasmacytoid dendritic cell
IFN: Interferon
IL: Interleukin
pPPARγ: Phosphorylated peroxisome proliferator-activated γ
pSTING: Phosphorylated stimulator of interferon genes
IHC: Immunohistochemical
IF: Immunofluorescence
IMC: Image mass cytometry
MPI: Mean pixel intensity
