# Correction to: Cannabinoid type 2 receptor (CB2R) distribution in dermatomyositis skin and peripheral blood mononuclear cells (PBMCs) and in vivo efects of Lenabasum^TM^

**DOI:** 10.1186/s13075-022-02758-1

**Published:** 2022-03-11

**Authors:** Spandana Maddukuri, Jay Patel, De Anna Diaz, Kristen L. Chen, Maria Wysocka, Christina Bax, Yubin Li, Adarsh Ravishankar, Madison Grinnell, Majid Zeidi, Nithin Reddy, Josef Symon S. Concha, Muhammad M. Bashir, Joyce Okawa, Barbara White, Victoria P. Werth

**Affiliations:** 1 Department of Dermatology, Corporal Michael J. Crescenz Veterans Affairs Medical Center, Philadelphia, PA USA; 2 Department of Dermatology, Perelman School of Medicine, University of Pennsylvania, Philadelphia, PA USA; 3 Department of Pathology, SUNY Downstate Health Sciences University, Brooklyn, NY USA; 4 Department of Medicine, Division of Dermatology, Albert Einstein College of Medicine and Montefiore Medical Center, Bronx, NY USA; 5 Corbus Pharmaceuticals, Inc., Norwood, MA USA

**Correction to: Arthritis Res Ther 24, 12 (2022)**

**https://doi.org/10.1186/s13075-021-02665-x**

Following publication of the original article [1], the authors identified an error in Fig. 3. The correct figure is given below.

**Fig. 3:**
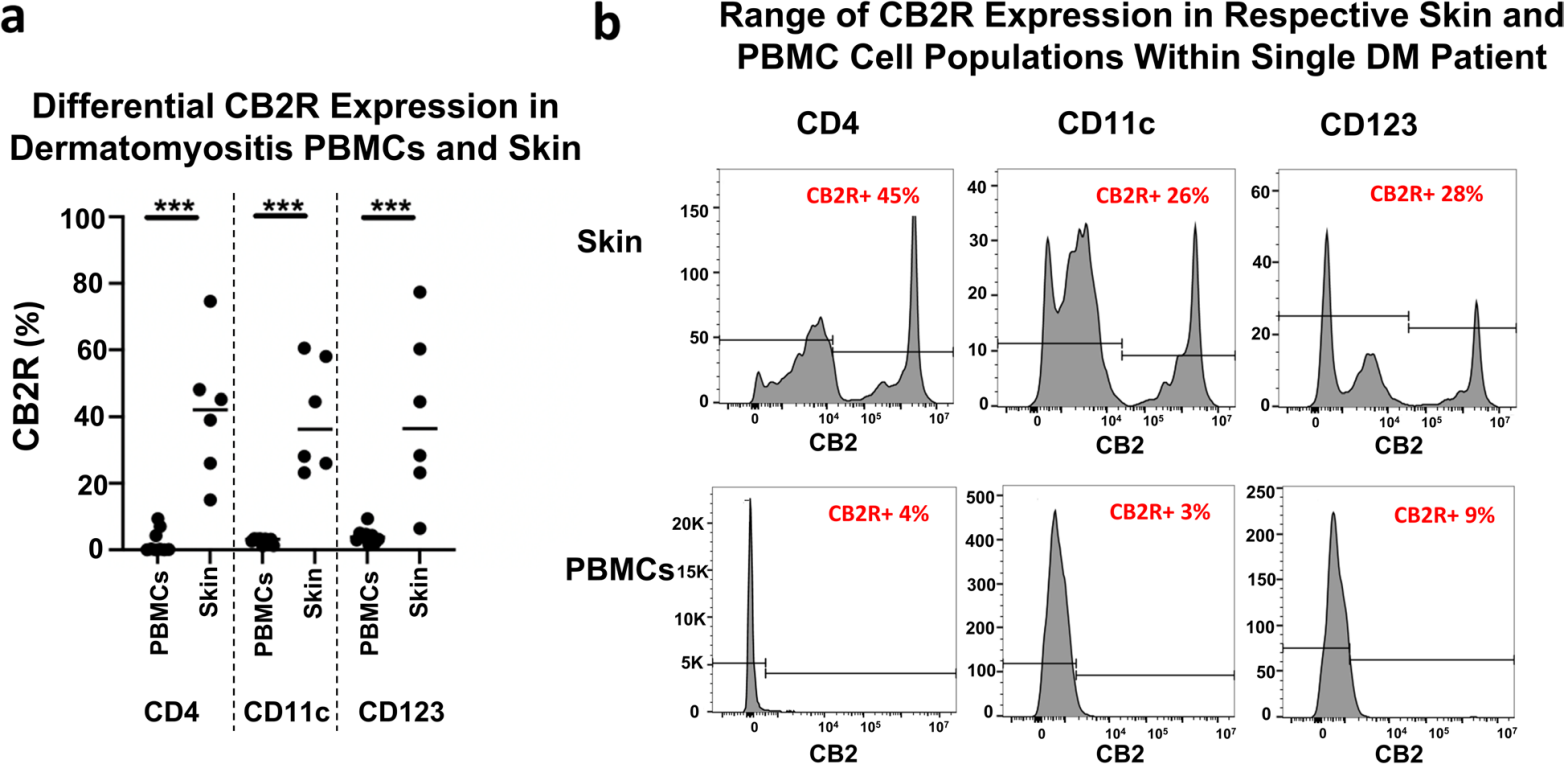
CB2R distribution among DM skin and DM PBMCs via flow cytometry. DM and HC peripheral blood PBMCs and skin cells isolated and stained via flow cytometry. a Percentage of CB2R expression in CD4+ T cells, CD11c+ mDCs, and CD123+ pDCs compared between DM peripheral blood and skin. b CB2R expression in skin and peripheral blood samples of a single patient shown with respect to CD4+ T cells, CD11c+ mDCs, and CD123+ pDCs. Graph shows the median. ***p<0.001. DM, dermatomyositis; HC, healthy control; mDC, myeloid dendritic cells; PBMC, peripheral blood mononuclear cell; pDC, plasmacytoid dendritic cell

The original article [1] has been updated.
