# Supplementary material for: Cannabinoid type 2 receptor (CB2R) distribution in dermatomyositis skin and peripheral blood mononuclear cells (PBMCs) and in vivo effects of LenabasumTM
Source: Arthritis Res Ther. 2022 Jan 4;24:12. doi: 10.1186/s13075-021-02665-x (PMC8725283; doi:10.1186/s13075-021-02665-x)
Supplement: Supplementary file 1 — Additional file 1. Supplementary methods [file 13075_2021_2665_MOESM1_ESM.pdf]

SUPPLEMENTARY METHODS:

Immunohistochemical staining

Formal-fixed and paraffin embedded (FFPE) 4-mm skin biopsies were cut into 5-μm sections and placed onto glass slides. Slides were dewaxed at 60ºC overnight and rehydrated subsequently by washing in Citrisolv® (Fisher Scientific, Waltham, MA), serial ethanol dilutions, and deionized water (DI) water. Heat-induced antigen retrieval was performed in EDTA in a 95 ºC pressure cooker. Sections were blocked at room temperature (RT) for 4 hours with 5% bovine serum albumin (BSA) and incubated with primary antibody at 4°C overnight. Tissues were stained for CD4+ T cells, IFN-β, IFN-γ, IL-31, CB2R, CD11c, and CD123. Antibodies and dilutions are detailed in Table S5. Samples were probed with biotinylated anti-goat/anti-mouse/anti-rabbit (Abcam, ab64257) for 50 minutes and streptavidin-HRP (Dako) for 30 minutes. Tissues were developed using NOVAred® chromogen (Vector Laboratories, Burlingame, CA) for 5-10 minutes. Tissues were dehydrated using ethanol serial dilution and Citrisolv®, then mounted with Permount (Fisher Scientific) and glass coverslips. Sections were analyzed with the Nikon Eclipse 80i microscope (Nikon, Tokyo, Japan). Cells in the dermis were quantified by taking an average of five nonoverlapping 40x objective magnification fields. Cytokine staining was quantified via percent of area stained using NIS Elements software in one 20x magnification field.

Immunofluorescence Staining

Initial slide preparation was completed as delineated in immunohistochemistry staining methods. However, for immunofluorescence, sections were blocked at room temperature for 2 hours with 3% bovine serum albumin (BSA). Tissues were thereafter incubated in primary antibodies (Table

S5) overnight at 4ºC. After washing with phosphate-buffered saline/tween (PBST), secondary antibody incubation was done at RT for 1 hour with appropriate goat anti-mouse/rabbit antibodies conjugated to Alexa Flour 488/594 (Thermo Fisher, Waltham, MA). Tissues were washed with PBST and treated with TrueView (Vector Laboratories, Burlingame, CA) for 2 minutes after which they were washed and mounted with Vector antifade mounting medium with DAPI.

Image Mass Cytometry

Antibody Staining and Image Acquisition

Tissue processing proceeded s previously described, however after blocking at RT with 3% BSA for 2 hours, tissue sections were incubated in a cocktail of 17 metal conjugated antibodies in phosphate-buffered saline (PBS) with 1% BSA overnight at 4ºC. Appropriate dilutions and antibody information are detailed in Table S6. The next day, slides were washed in PBST and incubated with a 1:400 dilution of Intercalator-Ir (Fluidigm-201192B, San Francisco, CA) for 30 minutes at RT. Slides were washed 2 times in DI water for 2 minutes each and air dried for 30 minutes afterwards.

RNA extraction and quantitative real-time reverse transcriptase polymerase chain reaction (QRT-PCR)

RNA from 12 formalin-fixed, paraffin-embedded (FFPE) lesional skin biopsies from the lenabasum trial were extracted using the RNeasy FFPE Kit (Qiagen, Valencia, CA). All RNA samples were converted to cDNA using the High-Capacity cDNA Reverse Transcription Kit (Applied Biosystems, Foster City, CA) for subsequent use in PCR. Gene expressions in skin

tissue were measured by performing QRT-PCR on cDNA samples using a TaqMan custom designed array card assay (Applied Biosystems, Foster City, CA) per manufacturer's protocol. The following probes were used: Applied Biosystems; IFNG, Hs00989291_m1; CMPK2, Hs01013364_m1; CXCL10, Hs00171042_m1; HERC5, Hs00180943_m1; IFI27, Hs01086373_g1; IFI44, Hs00197427_m1; IFI44L, Hs00915292_m1; IFI6, Hs00242571_m1; IFIT1, Hs03027069_s1; ISG15, Hs01921425_s1; RSAD2, Hs00369813_m1. The assay was performed on the VIIA 7 Real-Time PCR (Applied Biosystems, Foster City, CA). Gene transcript levels were normalized to human GAPDH (Applied Biosystems, Hs99999905_m1) and relative gene expression was calculated using the comparative CT method (ΔΔCt). The IFN-β signature was calculated by obtaining the median relative expression of 10 genes previously known to correlate strongly with serum IFN-β levels (Huard 2017): CMPK2, CXCL10, HERC5, IFI27, IFI44, IFI44L, IFI6, IFIT1, ISG15, and RSAD2.

Flow Cytometry

Stimulation of Cells, Staining, and Acquisition of Data

For lymphocyte samples, $1x10^6$ cells were aliquoted into each FACS tube and, whereas $2x10^6$ cells were aliquoted for dendritic cell samples. Samples were then pretreated with staining buffer (2% FCS), blocked with mouse IgG2b (Sigma Aldrich-I8765-10MG), and stained with surface antibodies (CD3, CD4, and CB2R for lymphocytes and Lineage cocktail, HLA-DR, CD11c, CD123, and CB2R for dendritic cells for the BD FACS Canto panels and CD45, CD3, CD4, CD8 and CB2R for lymphocytes and Lineage cocktail, HLA-DR, CD11c, CD123, and CB2R for dendritic cells for the BD FACSymphomy panels) for 30 minutes. Conjugated antibodies are

detailed in Table S7. Cells were washed in PBS, fixed and permeabilized, and stained for intracellular cytokines (IFNγ, IFNβ, IL31, and IL4 for lymphocytes and dendritic cells) for 20 minutes. Cells were resuspended in 0.2mL PBS and single-cell suspensions underwent flow cytometric analysis on a BD FACS Canto or BD FACS Symphony (BD Biosciences, San Jose, CA) and analyzed with FlowJo software (BD Biosciences). A total of 150,000 events were collected for analysis of lymphocytes and dendritic cells. Lymphocyte data obtained on the BD FACS Canto included gating on leukocytes by identifying CD3+ cells. CD8+ population was defined as being CD3+ and CD4-. Lymphocyte data obtained on the BD FACSymphony involved gating and identifying CD45+ cells and further identifying CD45+ cells expressing CD3. CD45+CD3+ cells were then stratified by CD4 and CD8 expression. Dendritic cells, from both staining panels, were identified by gating on leukocytes and identifying the HLA-DR+ and Lineage negative subpopulation. This excluded CD3+ T cells, CD14+/CD16+ monocytes/macrophages, CD16+/CD56+ NK cells, CD16+ neutrophils, CD19+/CD20+ B cells. The mDCs and pDCs were identified by CD11c and CD123, respectively.

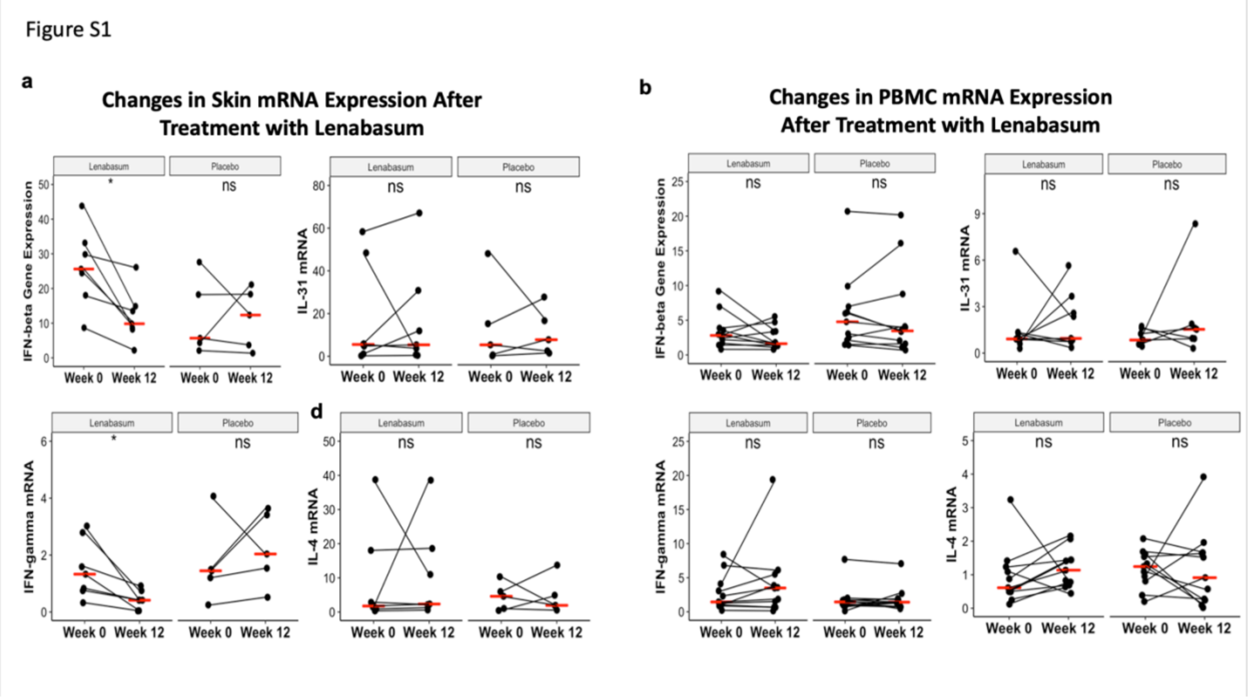

Figure S1. Cytokine mRNA expression in DM lesional skin and PBMCs treated with Lenabasum versus placebo

Real-time PCR was performed on mRNA extracted from DM (a) lesional skin biopsies and (b) PBMCs for IFN-β, IFN-γ, IL-31, and IL-4 at week 0 and week 12 following treatment with either lenabasum or placebo.

Supplementary Figure 2.

Gating by using FMO

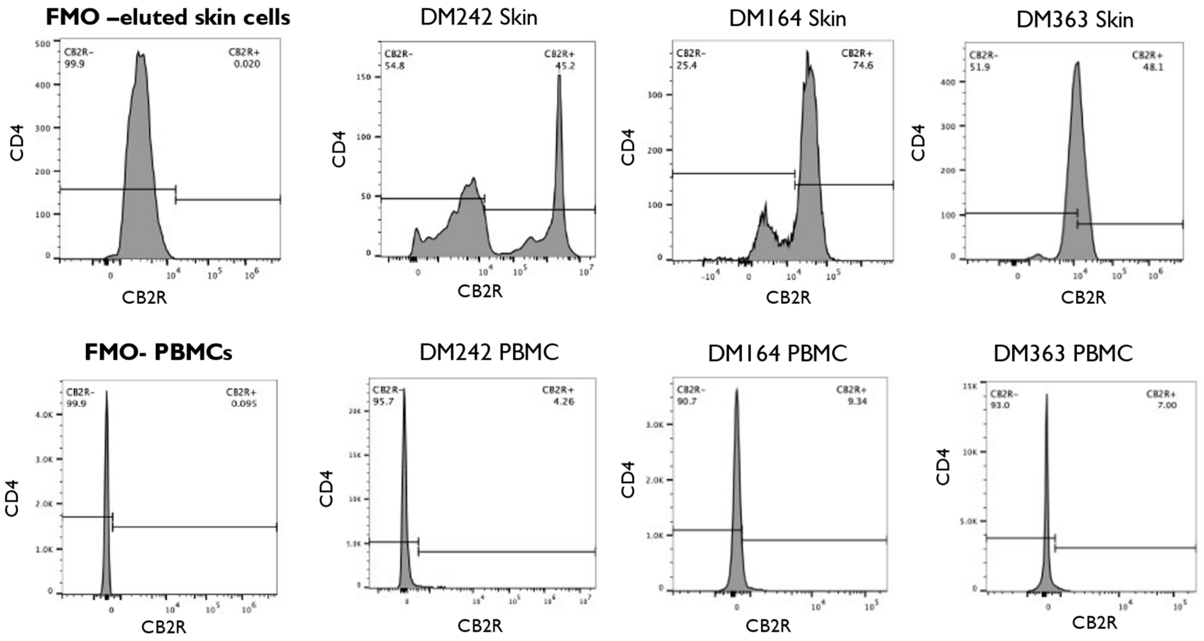

Gating by using FMO

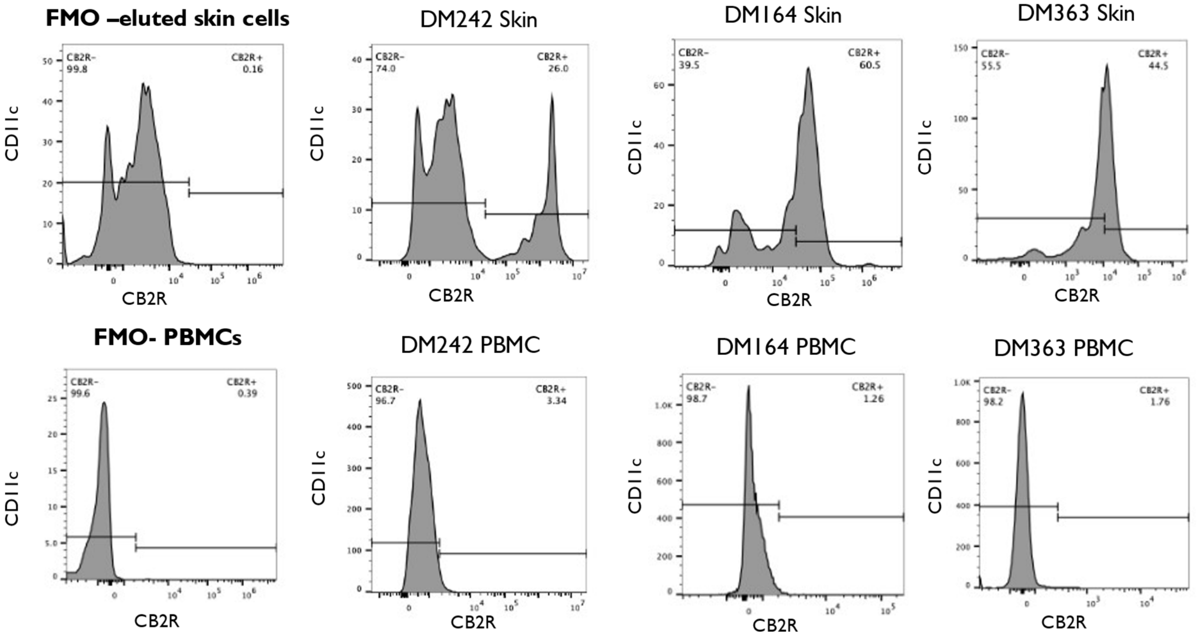

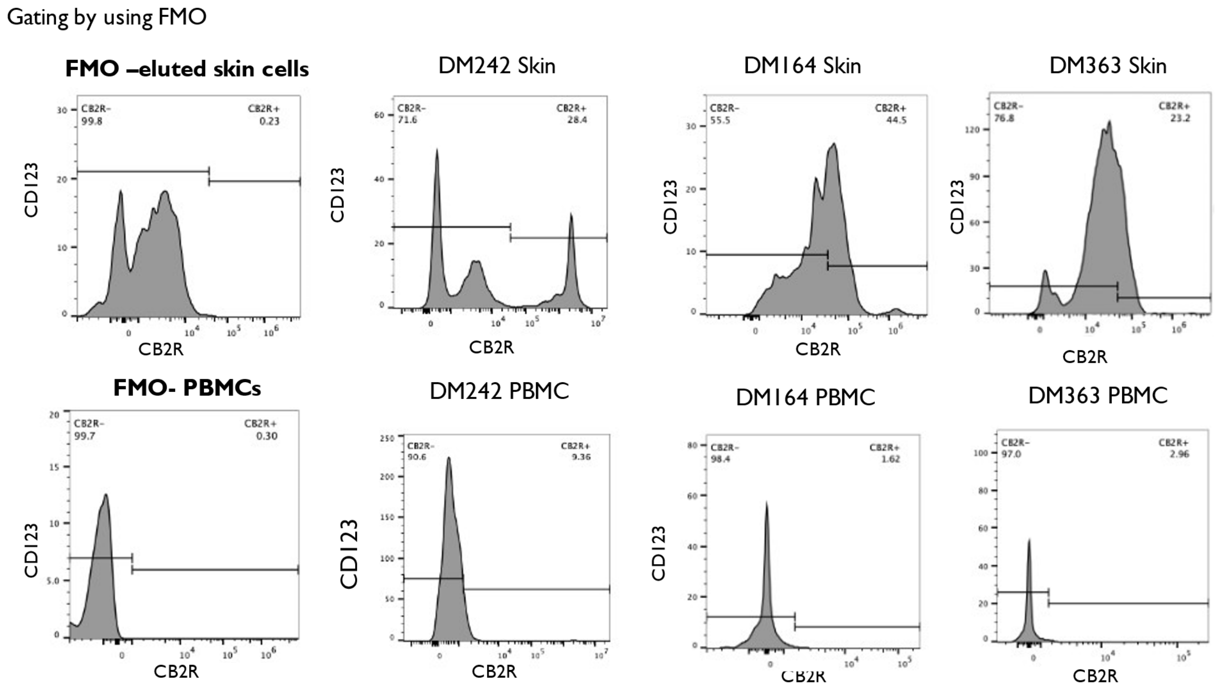

Figure S2. FMO controls for CB2R expression in CD4, CD11c, and CD123 cells

Flow Cytometry Data for three Dermatomyositis Patients eluted skin and corresponding PBMCs demonstrating use of FMO for CD4, CD11C, and CD123 to detect positive expression of CB2R.

Supplementary Figure 3.

CD4- DM164

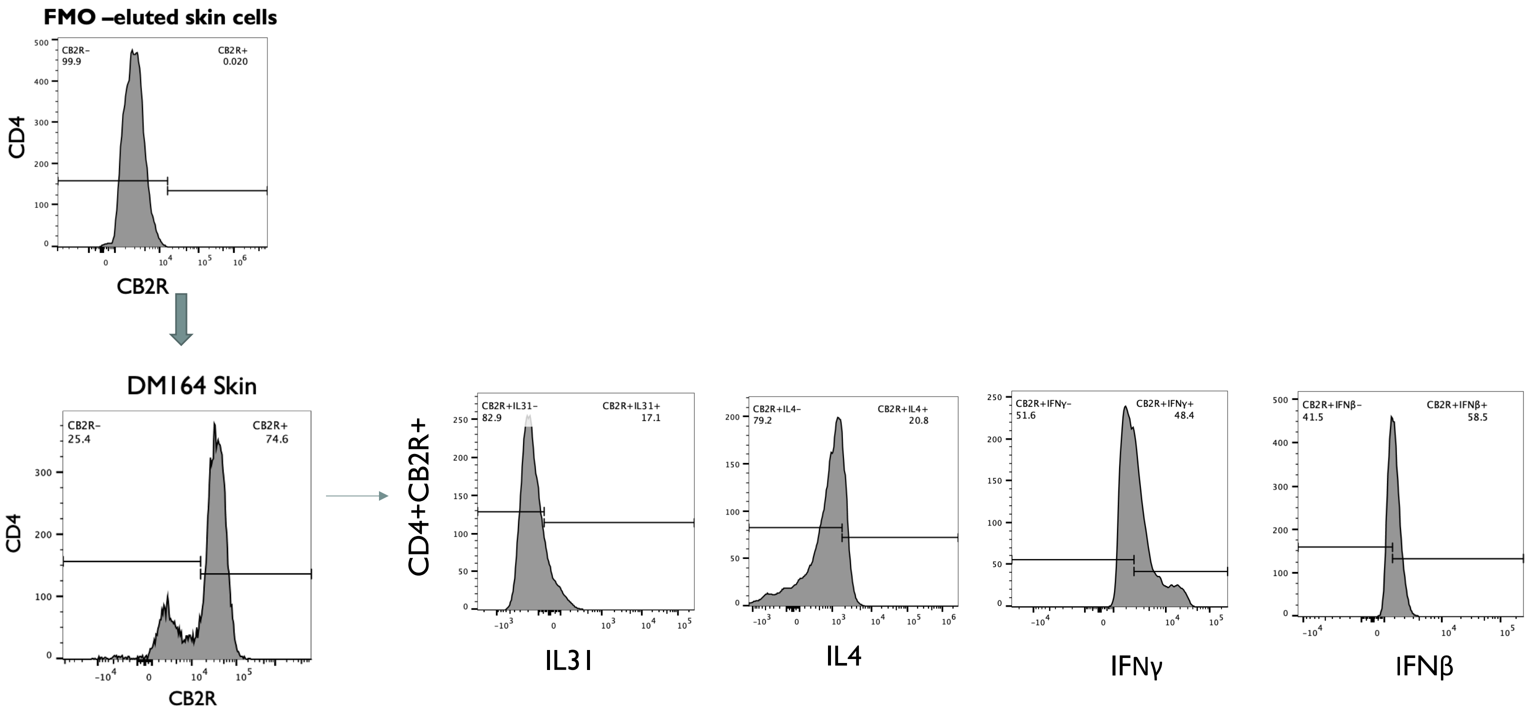

CD11c- DM164

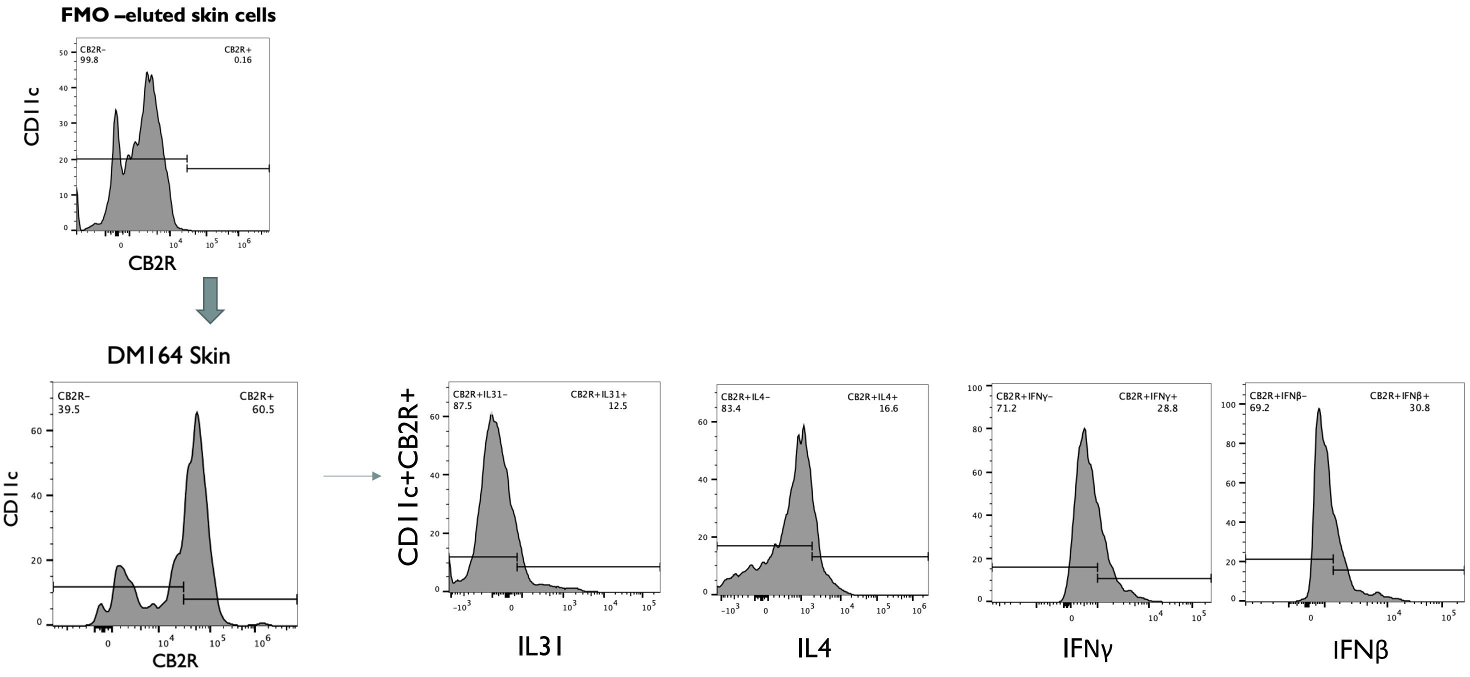

CD123- DM164

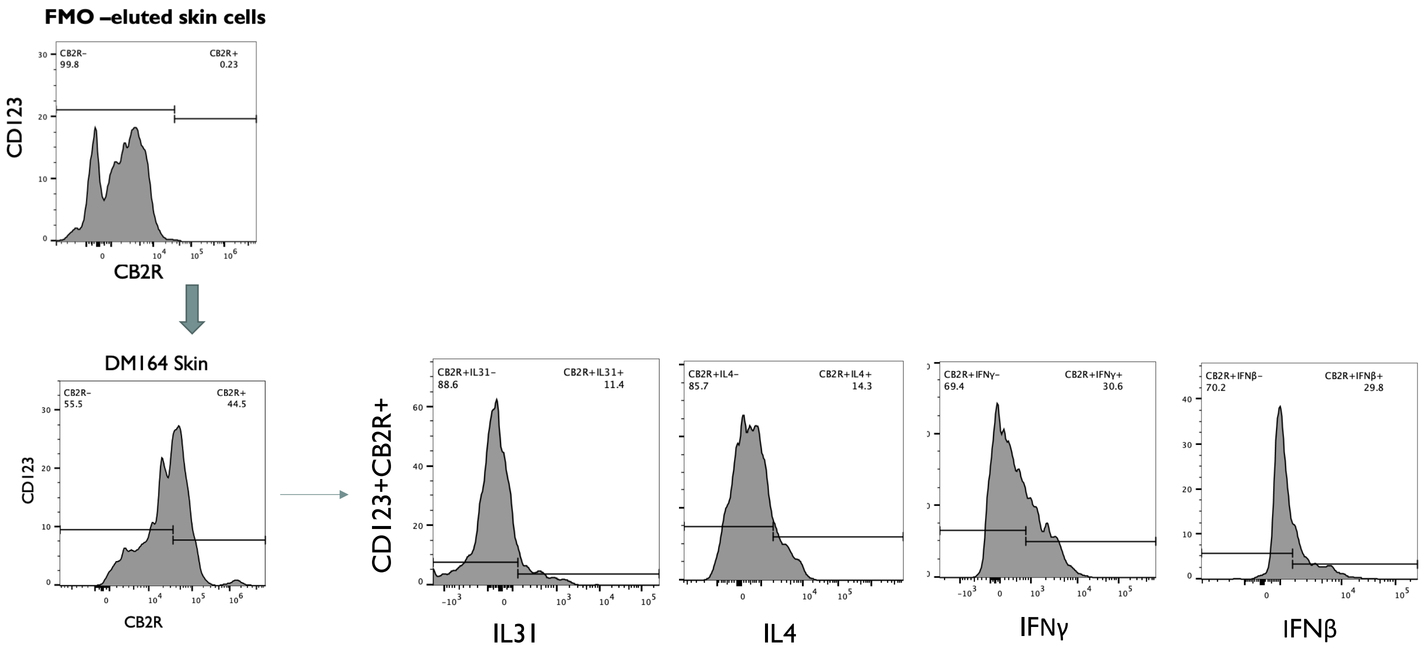

CD4- DM363

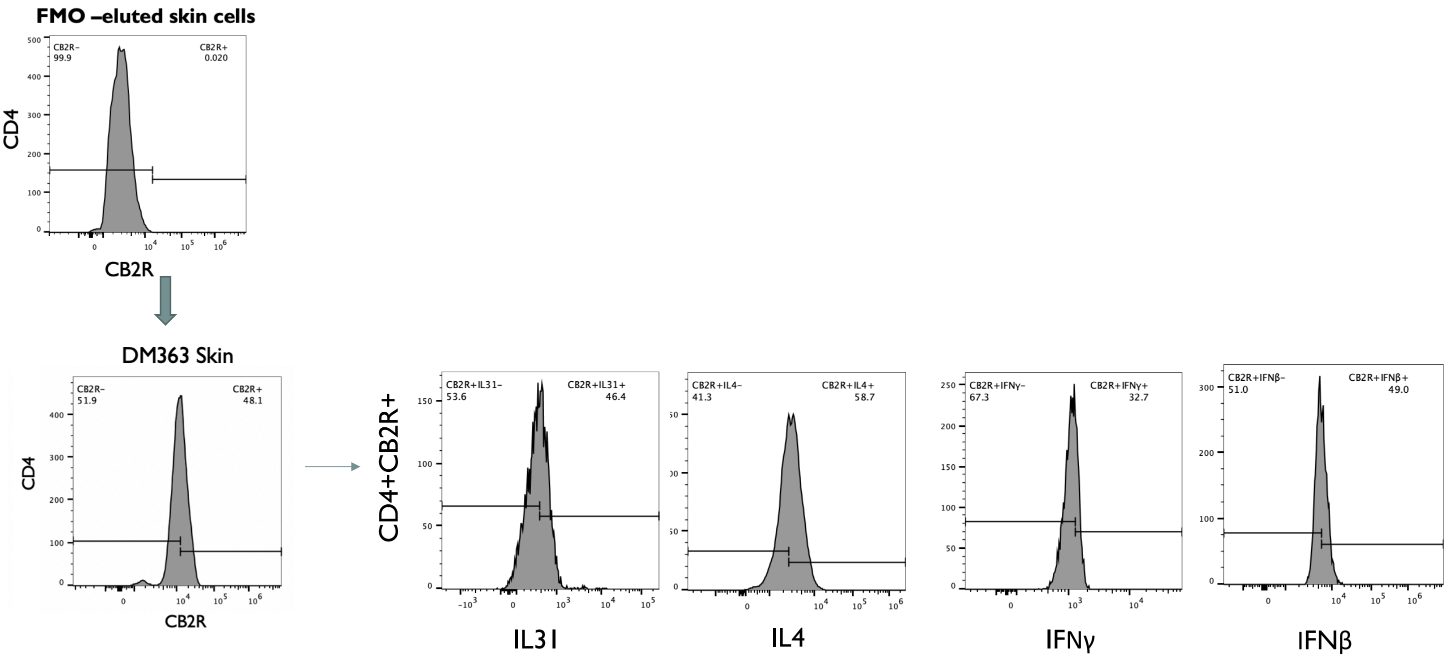

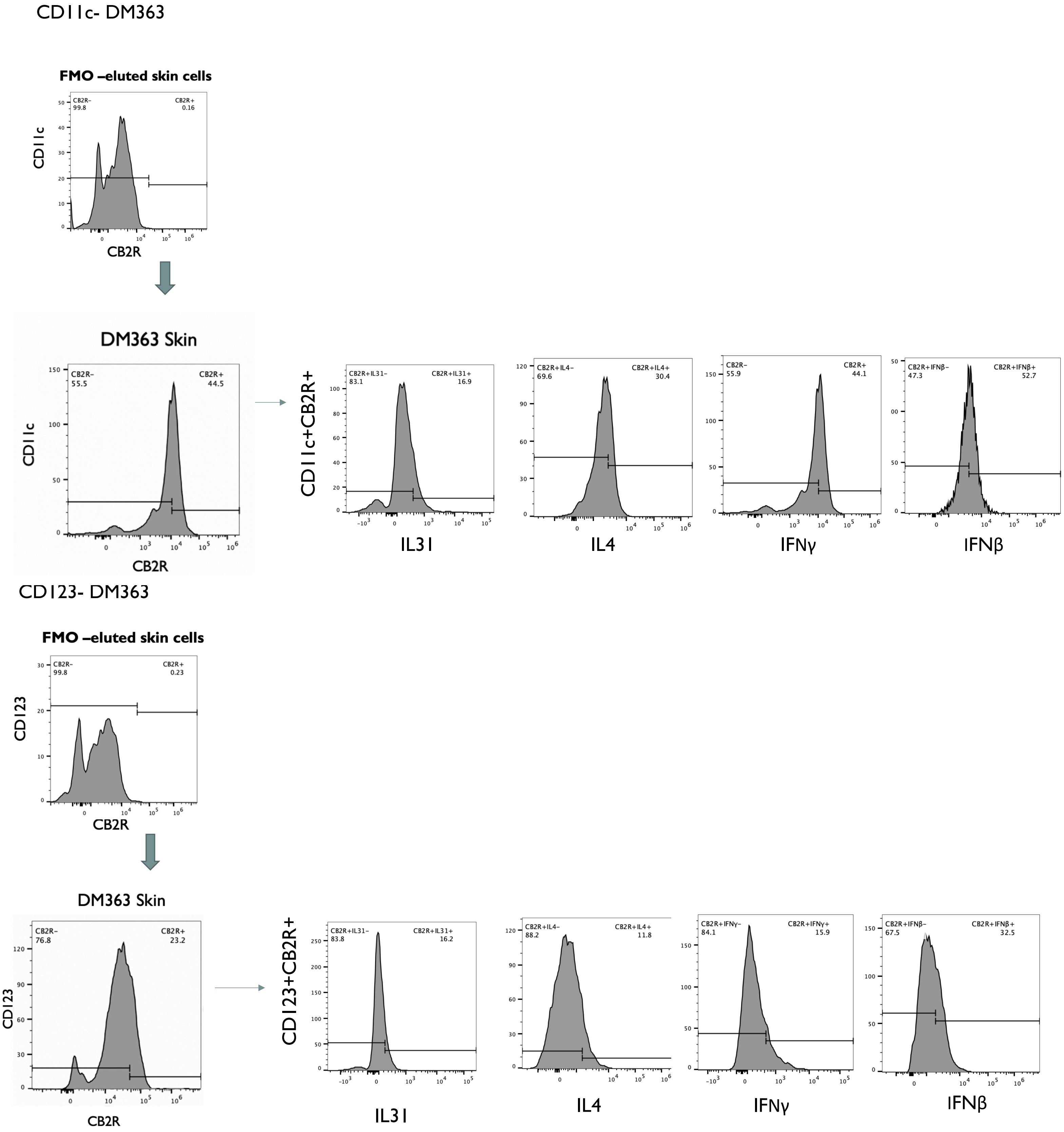

Figure S3. FMO and MFI based gating strategy of CB2R+ cells expressing IL31, IL4, IFNγ, and IFNβ

Flow Cytometry Data for three Dermatomyositis Patients eluted skin demonstrated FMO based CB2R cell positivity followed by detection of cytokines IL31, IL4, IFNγ, and IFNβ using MFI as described in methods.

SUPPLEMENTARY TABLES:

Table S1. Demographics for Immunohistochemistry and mRNA

	Lenabasum (n=7)	Placebo (n=5)
**Age (y), Median ± IQR**	54±16	57±16
**Sex, n (%)**		
Male	1 (14%)	0 (0%)
Female	6 (86%)	5 (100%)
**Race, n (%)**		
Caucasian	7 (100%)	4 (80%)
Non-Caucasian	0 (0%)	1 (20%)
**CDASI Activity Score, median ± IQR**	29±9	32±9.5

Table S2. Demographics for Image Mass Cytometry

	Healthy Controls (n=5)	Dermatomyositis (n=10)
**Age (y), Median ± IQR**	53.8 ± 6.7	56.7 ± 4.5
**Sex, n (%)**		
Male	0 (0%)	1 (10%)
Female	5 (100%)	9 (90%)
**Race, n (%)**		
Caucasian	5 (100%)	10 (100%)
Non-Caucasian	0 (0%)	0 (0%)
**CDASI Activity Score, median ± IQR**	N/A	27.5±9

Table S3. Demographics for Skin Biopsy Flow Cytometry (DM)

	Dermatomyositis (n=7)
**Age (y), Median ± IQR**	52.7±31
**Sex, n (%)**	
Male	1 (14%)
Female	6 (86%)
**Race, n (%)**	
Caucasian	7 (100%)
Non-Caucasian	0 (0%)
**CDASI Activity Score, median ± IQR**	21.6±9

Table S4. Demographics for PBMC Flow Cytometry

	Healthy Controls (n=11)	Dermatomyositis (n=19)*
**Age (y) Median ± IQR**	27 ± 5	62 ± 18
**Sex, n (%)**		
Male	8 (73%)	1 (6%)
Female	3 (27%)	16 (94%)
**Race, n (%)**		
Caucasian	4 (36%)	16 (84%)
Non-Caucasian	7 (64%)	3 (16%)
**CDASI Activity Score, median ± IQR**	N/A	9 ± 16

*2 DM patients were used twice at different time points for different experiments and were counted twice due to changed CDASI Activity Score at each time point.

Table S5. Primary antibodies and dilutions for immunohistochemistry and immunofluorescence

Antibody	Host Species	Dilution	Catalog # (Company)
CB2	Rabbit	1:50	LS-A34-50 (LsBio)
CD4	Rabbit	1:2000	ab133616 (Abcam)
CD11c	Rabbit	1:200	ab52632 (Abcam)
CD11c	Mouse	1:40	14-9761-82 (Thermofisher)
CD123	Mouse	1:25	6H6 (Cell Marque)
IFN-β	Rabbit	1:300	ab140211(Abcam)
IFN-γ	Mouse	1:200	ab218426 (Abcam)
IL-31	Rabbit	1:400	ab102750 (Abcam)

Table S6: Image Mass Cytometry Staining Panel

Antibody	CLONE	Channel	Dilution	Catolog # (Company)
CD20	H1	115In	200	555677 (BD)
pSTING	D8K6H	142Nd	30	40818S (CST)
CD14	EPR3653	144Nd	200	3144025D(Fluidigm)
CB2	polyclonal	147Sm	50	LS-A34-50 (LsBio)
CD31	EPR3094	151Eu	200	3151025D(Fluidigm)
FOXP3	236A/E7	155Gd	100	3155016D (Fluidigm)
CD4	EPRG855	156Gd	100	3156033D (Fluidigm)
CD68	KP1	159Tb	150	3159035D (Fluidigm)
BDCA2	992258	161Dy	30	MAB62991-100 (RD)
CD8	C8/144B	162Dy	200	3162034D (Fluidigm)
CD56	301021	164Dy	125	14255-1-AP (Proteintech)
CD3	polyclonal	170Er	100	3170019D (Fluidigm)

tryptase	AA11	172Yb	20000	ab2378 (Abcam)
CD11C	EP1347Y	173Yb	150	ab216655 (Abcam)
HLA-DR	TAL 1B5	174Yb	400	ab20181 (Abcam)
MAC387	MAC387	175Lu	800	ab22506 (Abcam)
CD163	5C6-FAT	176Yb	100	MCA1853(BioRad)

Table S7: Conjugated antibodies and dilutions for flow cytometry

**Antibody**	**Host Species**	**Experimental dilution (per 1 ml)**	**Catalog # (Company)**
Panels used for BD FACS CANTO			
Dendritic cell panel			
APC anti-human Lineage Cocktail	mouse	20 µl	348803 (Biolegend)
APC/Cy7 anti-human HLA-DR	mouse	3 µl	307618 (Biolegend)
PE/Cy7 anti-human CD11c	mouse	5 µl	337216 (Biolegend)
Alexa Fluor® 700 anti-human CD123	mouse	5 µl	306040 (Biolegend)
PerCP anti-human IFNγ	mouse	2 µl	502524 (Biolegend)
FITC anti-human IL4	mouse	2 µl	500807 (Biolegend)
PE anti-human IL31	mouse	1.5 µl	659604 (Biolegend)
Lymphocyte panel			
FITC anti-human CD3	mouse	3 µl	344804 (Biolegend)
APC/Cy7 anti-human CD8	mouse	3 µl	344714 (Biolegend)
Alexa Fluor® 405 anti-human CB2R	mouse	3 µl	FAB36551RV-100UG (NovusBio)
APC anti-human IFNγ	mouse	1 µl	502512 (Biolegend)
PE/Cy7 anti-human IL4	mouse	0.5 µl	500823 (Biolegend)
PE anti-human IL31	mouse	1 µl	659604 (Biolegend)
Panels used for LSR			
Dendritic cell panel			
APC anti-human Lineage cocktail	mouse	20µl	348803 (Biolegend)
PerCP-Cy5.5 anti-human HLA-DR	mouse	5µl	307630 (Biolegend)

PE-Cy7 anti-human CD11c	mouse	5µl	301608 (Biolegend)
BV711 anti-human CD123	mouse	5µl	306030 (Biolegend)
AF700 anti-human IL31RA	rat	2µl	FAB2769N-100ug (R&D Systems)
AF405 anti-human CB2R	mouse	3µl	FAB36551V-100ug (R&D Systems)
FITC anti-human IFNβ	mouse	0.5µl	21400-3 (Pbl Assay science)
BV650 anti-human IFNγ	mouse	1.5µl	502538 (Biolegend)
BUV737 anti-human IL-4	rat	2µl	612835 (BD Biosciences)
PE anti-human IL31	mouse	1µl	659604 (Biolegend)
Lymphocyte panel			
BUV563 anti-human CD45	mouse	2µl	748720 (BD biosciences)
BV605 anti-human CD3	mouse	2µl	317322 (Biolegend)
PE-Texas Red anti-human CD4	mouse	2µl	MHVD0417 (ThermoFischer Scientific)
BV786 anti-human CD8	mouse	2µl	563823 (BD Biosciences)
AF700 anti-human IL31RA	rat	2µl	FAB2769N-100ug (R&D Systems)
AF405 anti-human CB2R	mouse	3µl	FAB36551V-100ug (R&D Systems)
FITC anti-human IFNβ	mouse	0.5µl	21400-3 (Pbl Assay science)
BV650 anti-human IFNγ	mouse	1.5µl	502538 (Biolegend)
BUV737 anti-human IL-4	rat	2µl	612835 (BD Biosciences)
PE anti-human IL31	mouse	1µl	659604 (Biolegend)
